# A landscape analysis of psychedelic facilitation training in the US

**DOI:** 10.1371/journal.pone.0350037

**Published:** 2026-05-29

**Authors:** Roman Palitsky, Caroline Peacock, Jeffrey A. Breau, Paul Gillis-Smith, Gosia Sklodowska

**Affiliations:** 1 Emory Spiritual Health, Woodruff Health Sciences Center, Emory University, Atlanta, Georgia, United States of America; 2 Emory Center for Psychedelics and Spirituality, Atlanta, Georgia, United States of America; 3 Department of Psychiatry and Behavioral Sciences, Emory University School of Medicine, Atlanta, Georgia, United States of America; 4 Winship Cancer Institute, Emory Healthcare, Atlanta, Georgia, United States of America; 5 Center for the Study of World Religions, Harvard Divinity School, Cambridge, Massachusetts, United States of America; Universiti Sains Malaysia, MALAYSIA

## Abstract

Skilled interpersonal support and safety monitoring can enhance the therapeutic potential of psychedelic compounds and reduce the potential for harm. In the anticipation of legalized psychedelic care, and the recognition of ongoing psychedelic use across a variety of contexts, numerous training programs have been established to educate psychedelic facilitators in supporting individuals who take psychedelics. However, psychedelic facilitation training in the US has not been well characterized, making it difficult to appraise the strengths, needs, and gaps in this emerging discipline, especially with regard to the development of competencies in spiritually responsive care. This project used a quality improvement approach to identify common priorities, practices, needs, and gaps in the emerging field of psychedelic facilitation training from the standpoint of 13 established and emerging training organizations from the US. Interviews were conducted with one or more representatives from each participating organization. Notes from these interviews, once confirmed by the interviewees, were synthesized to identify common practices, procedures, priorities, and gaps. Areas of focus among the programs included: careful selection of trainees and faculty; content emphases in instruction; addressing spiritual, existential, religious, and theological topics; and teaching strategies used in programs. Several gaps were also identified, pointing to the need for continuing education among program graduates, parity with other disciplines, and development of field standards in training facilitators. Psychedelic facilitation training is an evolving discipline. Training programs and trainees may benefit from greater collaboration, including ongoing exchange about best practices and adjustments to scientific, social, economic, and regulatory developments.

## Introduction

Public awareness and use of psychedelics have been on the rise across sectors and communities in the United States [1–3]. Psychedelics are a class of compounds that produce acute, transient, and profound changes in sensation, perception, cognition, and affect, and include substances such as psilocybin, lysergic acid diethylamide (LSD), and at times 3,4-Methylenedioxymethamphetamine (MDMA) [4]. Sustained impacts of psychedelics on wellbeing and mood, as well as spiritual, existential, religious, and theological (SERT: 5,6) outcomes, have been demonstrated across a growing corpus of research [7–9], which includes multiple Phase I-III clinical trials [10–12]. Nevertheless, alongside potential benefits psychedelics carry some risks for harm [13,14]. Adverse events may occur during dosing [15], and in the days, weeks, or months after dosing post-acute cognitive, mood, worldview, and SERT disruptions have been recognized [16,17]. Maximizing the benefits while minimizing any harms is of utmost priority. For this reason, and especially in therapeutic contexts, there is consensus that safety monitoring during psychedelic dosing and—ideally—interpersonal support in separate sessions before and after dosing is recommended practice [18,19].

Practitioners who provide support for others who take psychedelics are referred to by a range of titles (e.g., psychedelic therapists, guides, monitors, coaches, etc.), but will be here referred to as “facilitators.” Facilitators are expected to receive some degree of specialized training for their role, but the training programs that provide such education are heterogeneous in terms of their training emphases, structure, content, and pedagogy. Because psychedelics have been demonstrated to interact with various SERT characteristics, the capacity for attending to SERT concerns in culturally responsive ways, with competence to meet care seekers’ needs without imposing on their autonomy, is important to cultivate among psychedelic facilitators [5,20]. Training in SERT-competent care is an unmet need among many helping professions, such as psychotherapy [21,22]. Ensuring that this topic is addressed in the training of psychedelic facilitators is especially important given the observed SERT-related impacts of psychedelics.

### Background on psychedelic facilitation training: Guidelines, regulations, and a proliferation of training programs

Psychedelics are currently Schedule I substances and are therefore illegal for most uses under federal law. Nevertheless, the Food and Drug Administration (FDA) has granted breakthrough therapy designations to several formulations of psilocybin and MDMA, and New Drug applications are anticipated which, if successful, would result in the rescheduling of several psychedelics on the weight of evidence of their therapeutic efficacy and relative safety. Decriminalization efforts have been successful in several states and jurisdictions such as Colorado, New Mexico, and Oregon. In light of these ongoing changes, and with the prospect of a psychedelic treatment boom on the horizon, there has been considerable interest in working with psychedelics for therapeutic, spiritual, and recreational purposes among various professions ([23,24], c.f. [25]). This interest has been met with a proliferation of psychedelic facilitation training programs in the US. At the time of writing, Blossom Analysis listed 110 therapist training courses and Psychedelicscourses.com listed 76 psychedelic therapist training courses worldwide [26].

Corresponding with this growth—or preparation for growth—in the psychedelic care sector, several organizations and government entities have introduced standards for psychedelic facilitation training, although no universally accepted accreditation or license currently exists in the US. Oregon passed a ballot measure allowing the regulated provision of psilocybin services in 2020 (Oregon Revised Statutes, 475A). In 2023 the first psilocybin service centers opened, and Oregon began to accept applications for licensure to provide care at these centers. The Oregon Health Authority, Oregon Psilocybin Services, and the Higher Education Coordinating Commission all inform the requirements for psilocybin facilitator training as it pertains to qualifications for psilocybin service centers, with courses ultimately approved by the Oregon Health Authority (ORS 475A.380). Current Oregon training program requirements include a total of 128 hours of instruction across 9 curriculum content areas (Table 1, c.f. ORA 333 §333; *Scope of* *Practice (SOP) for Oregon Psilocybin Facilitators* [27]), with any learning not done in person required to include at least 50% synchronous online engagement. Colorado’s Natural Medicine Health Act, SB23-290, became effective in 2023. Under the Act, to offer license-eligible training in Colorado, programs must be certified by the Colorado Department of Regulatory Agencies, Division of Professions and Occupations. To do so, they must offer coursework comprising at least 150 hours across 14 topic areas (Table 1, c.f. Colorado Natural Medicine Health Act). License recipients must have existing licensure for clinical practice (e.g., MD, PA, LCSW). In 2025 the New Mexico legislature approved SB219, a bill that would allow medical use of psilocybin. However, rules concerning facilitator training program requirements have not yet been instituted at time of writing. Although no stipulations about training exist at the federal level, draft guidance for clinical research with psychedelic drugs was provided and opened for commentary by the FDA’s Center for Drug Evaluation and Research in 2023. Under this guidance, lead monitors are expected to be a “healthcare provider with graduate-level professional training and clinical experience in psychotherapy, licensed to practice independently” (FDA-2023-D-1987, p. 9–10).

**Table 1:** State guidelines for psychedelic facilitation training in Oregon and Colorado.

Oregon	Hours	Colorado	Hours
Historical, Traditional, and Contemporary Practices and Applications	12		
Cultural Equity in Relation to Psilocybin Services	12	Indigenous Social, and Cultural Considerations	10
Safety, Ethics, and Responsibilities	12	Ethics and Colorado Natural Medicine Rules and Regulations	25
		Physical and Mental Health and State	25
		Facilitator Development and Self-Care	
Psilocybin Pharmacology, Neuroscience, and Clinical Research	12	Drug Effects, Contraindications, and Interactions	5
Core Facilitation Skills	16	Facilitator Best Practices	5
		Relation Boundaries and Introduction to Physical Touch	10
		Introduction to Trauma Informed Care	10
		Introduction to Suicide Risk	5
Preparation and Orientation	16	Screening	5
		Preparation	10
Administration	20	Administration	10
Integration	12	Integration	10
Group Facilitation	16	Group Facilitation	10
**Total hours**	**128**		**150**

Note: Content areas required for psychedelic facilitation training programs to be eligible for Oregon and Colorado certification are presented underneath column headers for each state, respectively. The number of hours required to be dedicated to each content area are listed to the right of each area. Content areas for each state are organized according to similarity. For example, requirements from each state pertaining to culture are located alongside one another. Where requirements from one state did not have counterparts in the other state, or when a single topic in one state corresponded to multiple topic in another state, fields in the table were left empty. When one state included multiple areas that all corresponded with a requirement for the other state, fields were left empty as well (e.g., OR’s “core facilitation skills” corresponded with multiple requirements in CO guidelines).

Several non-government entities have been influential for the development of training standards. In 2023 the American Psychedelic Practitioners Association (APPA, formed 2021, dissolved 2023), in collaboration with Brainfutures, presented their Professional Practice Guidelines for Psychedelic-Assisted Therapy [28]. These general guidelines included direction that psychedelic-assisted therapy (PAT) practitioners should have appropriate training, which included “potential physical, psychological, and spiritual effects of psychedelics and core principles of psychedelic therapy” (Guideline 2). The guidelines do not offer specific criteria or recommendations in the way that subsequent state guidelines have. However, unlike state guidelines the APPA/Brainfutures guidelines explicitly mention spirituality as an area of competence for practitioners because, according to the document, “psychological, as well as spiritual or existential, effects can be quite unique to psychedelic-assisted therapy” (p. 14).

### Landscape analysis

Understanding the landscape of psychedelic facilitation training, identifying existing educational programs’ priorities for training, and addressing gaps—especially insofar as SERT-competent care is concerned—are priorities for this emerging field. However, the prevailing practices in psychedelic training are moving targets. Depending on the state of regulatory, educational, economic, and social environments, programs are founded or go out of business, change acceptance criteria for trainees, shift training emphases and priority trainee groups, and alter locations within or outside of the US. Several groups continually track and report on extant programs in and out of the US, providing a broad overview of available educational activities related to psychedelic facilitation [26,29]. To supplement currently existing catalogs of psychedelic training programs with an in-depth assessment of training priorities and practices, this project conducted a series of interviews with representatives from training programs in the US that serve different subsectors within the field.

This project sought to gather information about psychedelic facilitation training from established and emerging leaders in that field. Our first aim was to gather information about the landscape of practices and priorities of psychedelic facilitation training, and to better understand how programs navigate the real-world exigencies of their training contexts while seeking to provide quality training. Our second aim was to identify the unmet needs, or gaps, in psychedelic facilitation training. Finally, we aimed to identify potential recommendations to address these needs. Across these aims, our scope included a focus on SERT-responsive care training, as well as domains relevant to spiritual care or chaplaincy, to examine how this recognized need in psychedelic care is addressed in facilitator training.

## Method

### Project development

This project first developed a quality improvement (QI) research strategy through a series of biweekly discussions among the research team over 2 months. Topics of interest that could extend knowledge about current practices in psychedelic facilitation training were developed during these team meetings and by consulting with outside experts. Rather than testing hypotheses or aiming at generalizability, this project is intended for the psychedelic facilitation training field to learn about itself, hone its priorities, and provide summative information to the broader scientific and pedagogical communities interested in this topic. As a quality improvement project that did not provide an intervention or seek to generate generalizable data, this work was deemed non-human-subjects-research by the Emory University IRB.

### Recruitment

This project included programs that were: in the US, focused on training facilitators to work with classic psychedelics and MDMA but not ketamine, involved the training of facilitators rather than psychedelic education more broadly, were interactive (rather than learning management systems only), and focused on macrodosing rather than microdosing. This project did not include spiritual and cultural lineages that provide specialized training in the use of psychedelic plants and fungi, and which are not represented among training programs. The reason for this is that such training is often further-reaching than is expected from commercial psychedelic facilitation programs, and at the same time is not available to the public in the same way that a commercial training would be. Such teaching would require its own inquiry and is beyond the purview of this project. Snowball recruitment was used to contact psychedelic facilitation training programs. Although no formal quotas were used, purposive sampling aided the recruitment of facilitators representing a range of programs that had different explicit purposes, trainees, program ages, and contexts.

### Procedures

After contacting training programs and explaining the purpose of our project, we scheduled times to meet with representatives from those programs. Interviews were conducted via Zoom. Interviewees were sent information about the nature of the interviews in an email, which was again reviewed prior to the start of the interviews (see S1). Although need for consent was waived, we obtained verbal consent from all participants. We allowed participants to opt out of any part of this study, and to revise any information that we obtained from (see “member checking,” below). Measures to observe anonymity were explained to interviewees at that point as well. These included deletion of recordings after completion of summary notes, and not reporting specific information provided to us in ways that could be directly traced back to interviewees (i.e., reporting only in aggregate or anonymizing specific responses). Thus, it was explained that while the names of the programs would be retained, information would be reported such that it would not be possible to trace specific information back to any specific program (e.g., relying on ranges instead of specific numbers, or further obscuring information that might lead to the identification of specific programs through their reputations). Although not deemed human subjects research requiring consent, all steps consistent with informed consent were undertaken; consent was verbally obtained from all participants, and they were also explained that they could change or revise their information at any point that they chose. This included member checking, in which all participants were sent the notes synthesized from their interviews, which were then used as data for the report. Interviews were recorded with the explicit assent of interviewees. Recordings were not transcribed; they were only retained for verification of accuracy and then deleted. One interviewee preferred to conduct the interview via email and provided emailed responses to questions. Note-taking was otherwise used during interviews to collect information. These notes were then summarized into a synthesis of each conversation. As described above, these syntheses were then sent to each interviewee who participated via email with a request for confirmation or correction (i.e., member-checking). Three interviewees made corrections to the syntheses that we provided back to them, which included clarifications, the addition of information, or the correction of mistakes.

### Measures and materials

All data were collected in the form of interviews, which were designed around a rubric developed by the authors and with input from external advisors. The rubric was designed to reflect criteria deemed important for an understanding of psychedelic facilitation training. Rubric domains included structural, pedagogical, content, process, and philosophical aspects of training, as well as challenges and aspirations that interviewees observed within their own programs and in the field more broadly. The full interview guide is included in S1. The interviews were designed with the anticipation that each discussion would focus on a subset of elements within the rubric that a given program prioritized, or understood to be important, for psychedelic facilitation training. This means that frequencies of endorsement do not represent rates at which participating programs engage with any given practice or pedagogy. For example, if three programs’ representatives discussed the importance of cultural competence in training, it does not mean that the other programs do not provide such training, only that the other programs prioritized other elements during the interviews. The only exception to this procedure was SERT-related material: this topic was addressed with every program.

### Analysis

After member-checking, all summaries were synthesized within a single document that described and organized all of the information obtained from individual interviews in aggregate. Synthesis was conducted by the first two authors and guided by an inductive-deductive process: deductive procedures were governed by the structure of the interview rubric, and therefore by the research team’s *a priori* views and questions concerning psychedelic facilitation. The inductive procedures sought to include and organize informative statements by interviewees that generated novel topics or content beyond the initial rubric. This synthetic document was then reviewed by the first two authors alongside each individual interview summary to ensure fidelity, and protection of the identities of programs that provided specific responses. The summary document produced information about (1) the current context of psychedelic facilitation training, including emphases and norms; and (2) gaps in the psychedelic facilitation training, especially with regard to spiritual care. This included (2a) the challenges that program representatives shared from their own programs’ experiences, as well as for the field as a whole, and (2b) gaps that the authors identified by reviewing the findings of this project.

## Results

Interviews were conducted between December 2024 and April 2025. 20 programs were contacted to interview, 13 (68%) responded affirmatively and were interviewed. These included: Berkeley Center for the Science of Psychedelics, California Institute for Integral Studies, Embark, GuideSite, InnerTrek, Multidisciplinary Association for Psychedelic Studies, Myco Method, Oregon Psychedelic Institute, Subtle Winds, Synaptic/Bastyr Psychedelic Training (at the time of interview, Synaptic Institute had discontinued operations and was in process of transition to become integrated with Bastyr University’s facilitation training program), Usona Institute, and Vital. Interviews with four programs were conducted over two meetings instead of one due to time constraints. Per our commitment to present information only in aggregate rather than about any one program specifically, characteristics of training programs are included in summary form to represent the range of programs sampled in Table 2.

**Table 2:** Summary Descriptions of Included Programs’ Characteristics.

Length of training	Ranged between single-day immersive modules and multi-year programs that include mentored practica and ongoing supervision or consultation
Hours in training	12–185+ (including ongoing supervision and practica)
Cohort size	5–100 + trainees per cohort
Program history^a^	The youngest programs were mid-launch, while the oldest had training experience of over 10 years
Association with organizations and entities	Programs included: Independent, unaffiliated programs; programs affiliated with pharmaceutical organizations, programs affiliated with advocacy and scientific organizations, and university-affiliated programs.
Certification	Although multiple programs offered certificates, not all programs did, and some explicitly did not provide a certificate for practice. Some programs offered different levels of training, with only the most extensive training providing eligibility for a certificate.
Substance focus	Substance focus included psilocybin only, MDMA only, MDMA and psilocybin, and trans-drug psychedelic foci.
Practice focus	Training focused on a range of practice contexts: underground practice, harm reduction, clinical trials, and psilocybin service centers, as well as non-traditional applications such as entrepreneurship, were among the practice foci. Most programs did not specialize in one practice context, but rather took a generalist approach that was amenable to more than one context.
Adherence to external standards and boards	Although not all programs were structured in accordance with regulatory standards for license eligibility, many did. Those included eligibility for licensure in Oregon, Colorado, and additional eligibility in some cases for international practice standards (e.g., in Switzerland or Australia).
Cost	$0 – $11,100 (typical range: $7,000 – $10,500)^b^

Note: (a) Program histories include different iterations of contiguous trainings, even if held under different names or structures over time, in order to best represent length of experience and expertise in a given program. For example, if a program refined its pedagogy over many years and then became affiliated with a new organization under a new name in the past year, we did not deem it appropriate to only consider this program <1 year old for the purpose of this analysis. (b) Program costs were dynamic and actively in the process of changing for many of the programs at the time of interviews. Prices listed are for trainees, since costs paid by institutions (e.g., entities conducting clinical trials) were varied, contingent on a variety of factors, and not always publicly shared. The prices included here are also lower for several programs than what they had asked in prior years. Some programs included different modules at different price points, which sometimes needed to be combined in order to attain complete training with that program: prices here represent only individual module prices, since all possible trainings offered by a program were not always required in order to meet criteria for practice. Typical range was computed by excluding programs that charged less than $100 for trainings, and the costliest program.

### People first: Faculty and trainee characteristics

#### Recruiting trainees with adequate foundations for learning and unlearning.

***Trainee selection*.** Trainee inclusion characteristics were among the most-discussed topics among training programs. Although programs selected for different characteristics in their incoming trainees, most felt that selection of appropriate trainees was a high priority. Most programs sought trainees with post-graduate training of some kind. The rationale for this preference was that psychedelic facilitation involves a complex and highly nuanced skillset, which is difficult to impart even with the 150 educational hours required for Colorado licensure. Recruiting trainees who already had established skillsets and backgrounds in a prior chosen field, and which facilitation training might build upon, helped address this issue. Even for programs that required high formal standards, it was typical for exceptions to be made for non-traditional trainees who demonstrated experience and competence in a mode of care that was relevant to psychedelic facilitation (e.g., history of underground practice, or a background in a traditional or Indigenous approach to psychedelic care). Many of these programs admitted clergy or chaplains as qualified practitioners.

Educational criteria for admission were not uniform. One program accepted any trainees with a high school diploma. Some programs eschewed formal guidelines on ethical grounds. These included concerns about medicalization and gatekeeping in psychedelic facilitation that reinforced existing institutional authority while continuing to exclude aspiring trainees and experts from outside US educational institutions, such as those trained within Indigenous communities and lineages. Notably, during our study formal requirements were being lowered among multiple programs due to changes in the training market.

Several other priorities informed admissions. Most programs prioritized inclusion of trainees from diverse backgrounds. One program representative observed that providing care to diverse populations was a competency that could not be taught only through didactics and required being in training with others who were different from oneself. A diverse cohort created a shared space in which skills could be obtained through collaboration among trainees and faculty from different demographic, cultural, and religious or spiritual backgrounds. The majority of programs also discussed trainee characteristics that would enable them to provide psychedelic care. These included qualities such as reflexivity, equanimity, open-mindedness, relational competence, humility, maturity, and empathic capacity. Because trainees are sometimes expected to have non-ordinary state of consciousness (NOSC) experiences of their own, admitting trainees for whom taking psychedelics would be especially risky can be hazardous for the trainees. Several programs thus applied to trainee selection some of the criteria that might be used to determine patients’ eligibility for psychedelic care, such as mental health history and related risk factors. Admissions processes typically involved written applications and interviews. These were reviewed by program staff, external evaluators, and (in one case) an artificial intelligence-supported tool.

***Faculty selection*****.** Faculty ranged in size, from only one faculty member in the smallest program to teams comprising dozens of core and support faculty and staff among the largest. Approaches to selecting faculty were often based on (a) aspects of their identity and personal qualities (e.g., their reputation and existing impact in the field of psychedelic care) and (b) their credentials and formal background (e.g., specific licensure, education, or other explicit criteria). Faculty selection was often aligned with specific training aims. For example, instructors with research backgrounds were sought to provide training on research literacy, while MDs or PharmDs were sought to provide pharmacology training. Only two programs explicitly included chaplains or clergy among faculty, although several additional programs included clergy or chaplains as advisors in the development of their programs.

### Content emphases in instruction

**Fig 1:**
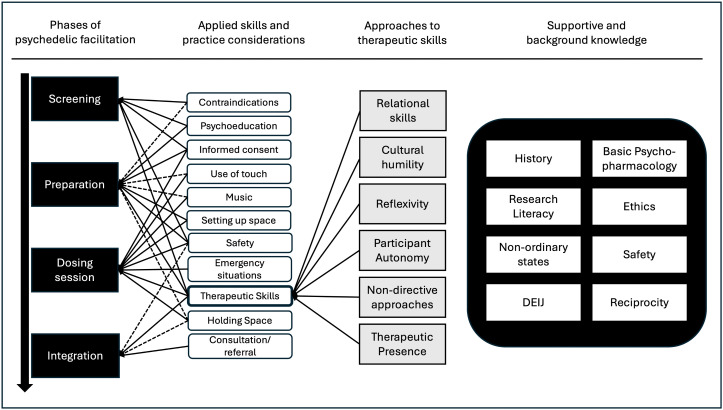
Content Emphases in Instruction. *Note*: This figure represents content emphases described by psychedelic facilitation training programs. Headings at the top of the figure refer to the content below each heading: The first, phases of psychedelic facilitation, represents in chronological order the phases of screening, preparation, dosing, and integration. Applied skills are illustrated in relation to the facilitation phases, with solid lines representing common, explicit references of a given skill to a given facilitation phase, while dashed lines represent only occasional mentions. Approaches to therapeutic skills are all included within “therapeutic skills”, which are an applied skill in facilitation, but which were discussed with depth and emphasis that warranted expansion as a discrete topic. Supportive and background knowledge includes aspects of instruction that were not explicitly tied to any given procedure but were taught due to their generalized importance for psychedelic care.

We include here topics that were repeatedly discussed by multiple programs as priority areas, or which represented similar topics that were synthesized into common themes (e.g., focus on diversity, representation, and social justice were brought together under the topic of diversity, equity, inclusion, and justice (DEIJ)). As represented in Fig 1, all programs discussed their training with reference to “phases” of psychedelic facilitation, which comprise screening, preparation, dosing, and integration. Specific elements of training that programs discussed were organized into three groups (discussed below, see also Fig 1): (1) applied skills and practice considerations; (2) approaches to psychotherapeutic skills (which emerged as a subset of the first category), and (3) supportive and background knowledge.

#### 1. Applied skills and practice considerations.

Fostering working knowledge for use in psychedelic facilitation was among the foremost priorities among training programs. This included knowledge about contraindications for psychedelics, especially during screening and preparation of clients, to ensure that psychedelics are not provided to those whom they might harm (e.g., those taking MAOIs), and that clients do not engage in activities that may negatively interact with subsequent use of psychedelics (e.g., problematic substance use). Several programs named ‘holding space’, which refers to providing a supportive presence without imposing on a person’s experience (c.f. [30]), as an important applied skill. Holding space was relevant to multiple aspects of facilitation but was primarily discussed in relation to the dosing session, and sometimes for offering supportive care during meaningful moments in preparation or integration phases. Other elements in this category included psychoeducation (though this term was not always used), informed consent (typically interpreted as an ongoing process), music, setting up the dosing space, safety considerations, appropriate response to emergency situations, appropriate consultation and referral, and “therapeutic skills.” Therapeutic skills were the subject of considerable expansion and detail, and were therefore expanded as their own category of content emphases, discussed below. Skillful use of touch was an explicit focus of training for multiple programs, often with instruction on what is and is not appropriate, what therapeutic touch entails, and informed consent models (e.g., [31,32]), although not all programs discussed this issue. There was a general interest, from programs that discussed touch, in having more structured resources and further knowledge sharing and evaluation of models for the appropriate use of touch.

#### 2. Therapeutic skills.

The *therapeutic skills* category in this analysis incudes any material that may be expected to fall under the purview of therapeutic practices, qualities, and activities. These comprise: relational skills, cultural humility, self-reflexivity, supporting participant autonomy, non-directive approaches (e.g., Rogerian or client-centered techniques [33]), and cultivation of a therapeutic presence (e.g., therapist’s use of self). Nearly all programs discussed the importance of trauma-sensitive care. Practices and principles that were occasionally mentioned but did not appear to be held in common across programs include psychodynamic principles such as transference and countertransference, the principle of an “inner healing intelligence” [34], and mindfulness instruction.

To the extent that programs discussed specific therapeutic models, the most commonly held perspective was that facilitation should be non-directive in nature, and that non-directive care should be taught to trainees. Correspondingly, Rogerian or Client-Centered therapy was a frequently endorsed approach, and occasionally also described as a pedagogical method as well. Harm reduction frameworks and principles [35] were repeatedly endorsed across programs. “Set and setting” principles [36] were taught across all programs. Several programs included didactics concerning Jungian psychological principles. Some also discussed transpersonal psychology in the tradition of Stanislav Grof’s work [37] and the Spiritual Emergence/y framework that emerged from it [38]. Spiritual emergence/y frameworks provide a conceptualization for non-ordinary states of consciousness, and especially for distressing mental states that can arise in the context of psychedelic or other psycho-spiritually impactful practices like meditation. These sometimes-profound changes to psychological states and functioning are understood to be aspects of a spiritual crisis (or emergence/y) which, according to these frameworks, have positive potential and may optimally be addressed via psychospiritual rather than psychiatric means [38]. Several programs included instruction on Internal Family Systems therapy [39] in their curricula.

Skills that fall within the purview of psychotherapy were not always described in terms of “therapy” per se. Some programs preferred not to frame facilitation in terms of psychotherapy, or indeed as any form of therapy. For some, Oregon’s standards, which do not classify facilitation as therapy, were directly relevant to this decision. Nevertheless, as one program observed, trainees often have implicit expectations that they will learn psychotherapeutic techniques, and some graduate with the assumption that they have, even when programs explicitly state that they do not teach psychotherapy.

#### 3. Supportive and background knowledge.

Some content areas were deemed important knowledge for a facilitator to have and regularly included in training. Many of these are linked with requirements for facilitation certification at state levels (Table 1). Most programs included instruction on the history of psychedelics, as well as the sociocultural and political contexts relevant to psychedelics. Principles of psychopharmacology for psychedelic compounds were included in curricula to provide an understanding of their classification, biological action, and contraindications. Research literacy, often conveyed by a biomedical research specialist, was included to prepare psychedelic facilitators to navigate the developing literature. Ethics instruction was emphasized by all programs, including general ethical principles (e.g., from psychology, medicine, or social work), as well as unique ethical considerations for psychedelic care. Physical, psychological, and ethical safety considerations were discussed. Ethics was the only category that emerged as applied *and* background knowledge, and in many cases was discussed as a bottom line for training: preparing facilitators to support the safety of their clients and provide ethical care was imperative. Special attention was given to consent, autonomy, and navigation of power dynamics in psychedelic facilitation.

All programs discussed NOSCs. Some used a predominantly didactic approach (e.g., readings and lectures). Others provided students with opportunities for their own experiences with a psychedelic or psychedelic-like substance, although most did not themselves facilitate this experience at the time of our interviews. Those that did not facilitate such experiences either informed students about legal opportunities to experience NOSCs (e.g.,. in OR, CO, or outside the US), partnered with groups that provided such experiences as “opt-in” opportunities, or preferred not to be involved in students’ decisions around NOSC-inducing experiences. Some used techniques such as Holotropic Breathwork [40] to give students the opportunity to experience NOSCs in a guided context. These experiences also enabled trainees to practice facilitation with one another.

DEIJ issues were discussed in different ways, including: fostering awareness of inequity; learning about the US war on drugs; histories of coloniality; focus on access and inclusion; preparation of trainees to work with diverse clientele; and understanding the role of factors such as collective trauma in the suffering of individuals who seek psychedelic care, and in the lives of the trainees themselves. Reciprocity was repeatedly named in these interviews, and was enacted through inclusion of Indigenous instructors on staff by some programs, through relationships with local communities, and through programmatically enacted principles (e.g., scholarships or activities to support disadvantaged communities).

### Addressing SERT

Although most programs acknowledged the importance of SERT, the majority did not dedicate any curriculum components to SERT-related topics. Lack of time and resources were the primary limiting factors, although lack of expertise or clarity on the best ways to teach about SERT-responsive care were also mentioned at times. Even programs that provided over 200 hours of instructional time and extensive asynchronous material related that there did not seem to be enough time in the curriculum to deliver an ideal amount of content. Because state standards mandate specific content areas (see Table 1), SERT topics were sometimes addressed under the auspices of required topics, primarily under cultural competence. Cultural competence included topics such as capacity for perspective-taking, empathy, avoiding cultural encapsulation, and cultural humility. These capacities were extended to religious and spiritual issues when SERT-responsive care was presented as part of cultural competence. Otherwise, SERT-responsive care was either provided through additional instruction requiring extra time, or omitted in favor of other teaching priorities. Some programs gave explicit reasons why SERT topics were not a focus of their training. For example, a concern was raised about teaching outside of the Oregon licensure recommendations, and taking care not to overstep one’s bounds as a facilitator under those licensure guidelines. Several programs named a distinction between spirituality and religion, and affirmed an interest in addressing spiritual needs while also expressing concern about explicit religious content in the facilitation dynamic, as well as potential for religious imposition in psychedelic care.

Programs that included SERT-related content did so in a variety of ways. Some included specific modules or throughlines in the training pedagogy that addressed spiritual, religious, or pastoral approaches, which at times also included Indigenous histories and practices. Some programs consulted with spiritual health professionals or clergy on curriculum development, although this did not always translate to the development of SERT-specific didactics. SERT material was sometimes presented through specific psychological and transpersonal-psychological approaches. Stanislav Grof’s work in transpersonal psychology [37], and the related frameworks of spiritual emergence/y [38], were mentioned several times. Spiritual emergence/y was also discussed on several occasions in relation to understanding and navigating challenges or adverse effects. Jungian psychology was also mentioned by several programs as a way to include SERT content by several programs. Several programs discussed SERT material primarily with regard to mystical experience frameworks [8,41]. Mindfulness was also mentioned in several instances as a way of engaging spiritual material in training. One program described a carefully developed relationship with a psychedelic religious community. They suggested holding that partnership up as a model, so that trainees might observe and replicate similar relationships as a way of integrating SERT into care.

Multiple programs indicated that the degree of SERT-related training that could be offered by facilitation certifications may not be adequate. As with other skills, this challenge was at times addressed by applying rigorous criteria to trainee selection, such that those who underwent training would already have some foundational skills in SERT-competent care.

### Teaching strategies

Pedagogical approaches varied considerably across programs. Some drew upon pedagogical theories and practices that were developed outside of psychedelics. Multiple programs did not reference specific pedagogic philosophies or theories, and instead described their approach through specific teaching methods (e.g., lecture, role-play). Most programs integrated different teaching methods, including lectures (used by all programs); trainee dyads (used by most programs); group-based learning (most programs), and role plays (all programs). Multiple programs used videos of facilitation, including optimal and problematic examples, for instruction. Some programs included creative projects, such as making visual art. Several programs included retreats, during which trainees and instructors could spend blocks of time together focusing on course material and group process. Several programs had offered opportunities experience a NOSC (e.g., holotropic breathwork, legal use of ketamine, or other NOSC experiences), during which trainees could also practice facilitation with one another, although such programming depended on the shifting legal and operational constraints on such practices. A subset of programs included a practicum after preliminary formal training. For some of these, completion of the practicum was necessary to receive final certification to practice facilitation (per program guidelines, but not necessarily per state guidelines or other legal statutes). Most programs encouraged ongoing consultation for graduated trainees, whether through a community of trainees or through outside resources. Trainees were evaluated using various methods, including assessment of written content, observation of role plays, and interviews. One program implemented assistance by generative AI for evaluations of recorded trainee role plays.

Most programs discussed the importance of focusing on process, rather than only content, in teaching. This included attending to the dynamics among trainees, and between trainees and instructors. Such process characteristics (e.g., remaining open, inquisitive, and collaborative while navigating unfamiliar content) were often a substantial focus of training. One program representative emphasized that unlearning was as important as learning, especially for healthcare professionals who have been extensively trained in directive approaches to care.

### Certifications

Programs had diverse approaches to certification. Not every program offered a completion certificate: for example, brief trainings offered to external entities, such as medical or research centers, were not necessarily accompanied by a certificate. For those that offered a completion certificate, this was not always the same thing as state-endorsed certification for practice: for these, a certificate of completion from an accredited program was necessary but not sufficient for practice. Some programs preferred not to offer certifications because this might mislead trainees to feel that they had endorsement by the program to begin practicing, and several program interviewees made clear that such decisions should not be made by a program independently but must be consistent with local statutes and global competencies. Some interviewees, especially from those programs that offered different levels of training (e.g., with vs. without a practicum component) were concerned that trainees might mistake preliminary training for sufficient training for practice, and for this reason preferred not to award certificates. At present, the decision to offer a certificate of completion appears to matter for state-endorsed programs, certificates from which may be used as part of a credential application for practice alongside other requirements (e.g., such as an exam, background check, and other criteria for psilocybin facilitator licensure in OR).

### Gaps in the current training landscape

#### Observations shared by program representatives.

Multiple interviewees expressed a need for greater cohesion and collaboration within the field of psychedelic training. There was interest in common standards generated by stakeholders from the facilitation training field itself, despite (or in addition to) the existence of US state-specific standards regulating training. Certification bodies such as the Council for Accreditation of Counseling and Related Educational Programs (CACREP) and the Board of Psychedelic Medicine were identified as important institutional assets for achieving this aim. Programs affirmed the importance of partnerships with other organizations, such as research institutions, pharmaceutical organizations, universities, and non-profit organizations. Such partnerships may help to support both the program and the partnering organization and can give trainees richer networks from which to draw opportunities for practice and further learning. When asked about professional organizations that could support meetings, journals, and information-sharing practices specific to psychedelic facilitation training, the programs we spoke with did not know of such initiatives outside of large-scale meetings such as Psychedelic Science, a conference that gathers every two years and includes stakeholders across the spectrum of psychedelic sectors. However, they voiced interest in what such organizations might offer for the maturation of psychedelic training practices. Multiple programs supported alumni networks, both formally and informally, to provide additional community to trainees.

Multiple programs expressed concern about the uncertainty of post-certification prospects for trainees. Many mentioned that the training market appeared to be over-saturated, and that inadequate practicum and practice opportunities represented a bottleneck in training. Absence of a clear pathway toward applying one’s training after program completion meant that fewer potential students were interested in pursuing costly training to begin with. Among those who did, training was an uncertain investment toward a career, imparting greater burdens for less affluent trainees. Multiple programs expressed optimism about potential legal approval of psychedelic treatments, which would provide vocational pathways to trainees. A constantly evolving legal landscape also meant that training programs were sometimes uncertain about which standards to build their curricula around, to best support their trainees in transitioning to practice. Several programs said it would be valuable to learn what graduates went on to do after completion of facilitation training, although this information was not generally available. Collecting these data would be a straightforward and highly useful initiative for the emergence of a psychedelic facilitation discipline.

Programs observed that time or resource constraints left gaps in training content or learning opportunities. Multiple programs stated that more time is needed for training, and several said that multi-year support for trainees after formal conclusion of training, including continuing education resources would be optimal. More nuanced teaching around skills related to touch in psychedelic facilitation, and the establishment of practices around the resolution of boundary violations, were identified as needs. The role or importance of personal experience with psychedelics for facilitation training is currently undecided [42–44]; several interviewees noted that it is important for psychedelic facilitators to have their own NOSC experience with a psychedelic in order to provide support to others who have such experiences. These instructors identified a lack of adequate opportunities for trainees to legally experience NOSCs, especially with psychedelics, as an obstacle to training. Relatedly, some noted that current “observation-only” practicum models, which primarily require students to only be observers prior to certification, are not ideal for clients’ preferences (i.e., having an “observer” present during the experience) or safety. Several programs described inadequate depth of training in SERT to be a limitation, as noted earlier.

## Discussion

Psychedelic facilitator education is a field in active development. Although training programs were highly diverse, teaching to different applications and trainee cohorts, program leaders approached facilitation training with tremendous care. Most programs provided extensive and detailed rationales for the pedagogic choices they made, drawing on histories, practices, and information from a variety of sources. This lent some commonality to the diversity of the field and created the impression of an emerging shared lexicon of practices and knowledge. These conversations also revealed a high degree of reflexivity and willingness to engage in self-critique, naming and actively discussing challenges and growing edges for the field and for interviewees’ own programs.

There is a need for greater shared structure, communication pathways, and opportunities to exchange, debate, and iterate upon teaching practices. However, creating such networks requires time, money, and personnel, which may be challenging in the current training climate. Financial sustainability—especially without ties to external institutions or support from donors—is a growing challenge as the number of training programs proliferates disproportionately to the opportunities available for practice. There is a recognized need for scalable mental health access [45], which has been extended to psychedelics as well [46,47]. Notwithstanding, several interviewees had reservations about the scaling of psychedelic training. One interviewee who had worked with different cohort sizes explicitly stated that scaling up may be unrealistic and even unethical. As Poppe et al. [48] have observed, patients receiving psychedelic treatments can be vulnerable in many respects, necessitating comprehensive training of psychedelic facilitators. Yet multiple interviewees expressed concern that typical training programs do not have adequate time to convey all the information that trainees need to receive for independent facilitation practice, especially to those without prior relevant background.

It is likely that specific training practices, facilitation techniques, and teaching contents will continue to be refined. Programs are willingly and actively iterating their own curricula and many conveyed to us a constant state of constructive revision over the aspects of training they were able to control. However, these conversations also shed light on the large influence of factors outside the curriculum that influence training. The legal status of psychedelics is easily the most influential of these. Several programs looked to the development of OR and CO standards as opportunities to engage in formative discussions with other programs and build shared principles. However other factors, such as anticipated insurance, payor, and reimbursement systems; relationships with other clinical disciplines; federal policies impacting public health, research, and the alleviation of health disparities; and the status of higher education were all invoked as major influences on the structures that support facilitation training. As noted earlier, most programs adopted a generalist approach, rather than a specialty in clinical, community, underground, or research settings. In this respect, adherence to state standards does not necessarily make such standards a focus, but rather helps programs adapt to different training aims among students.

Poppe et al. [48] proposed a typology of (1) medical, (2) mixed, and (3) non-medical models of training in their position paper on ethics in psychedelic training. Although we did not observe a clear delineation of approaches along those lines (all programs drew upon a range of elements and strategies), the intended applications of at least one program was primarily medical, while some were primarily (though not exclusively) focused on non-medical community settings. Most of the ethical issues observed by Poppe et al. (2025) were shared by the programs we spoke with, including concerns about issues with trainee personal experiences of psychedelics, or the need to prepare trainees to skillfully respond to the multifaceted vulnerability of patients receiving psychedelic care. Although several interviewees spoke about the importance of boundaries between faculty and students, the issue of “guruism” noted by Poppe (2025) and others (e.g., [49]) was not discussed as such. Indeed, programs had fairly diverse views on issues of religion, spirituality, and their integration with psychedelic facilitation training. Though not included in prior analyses, one of the programs expressed an important additional concern: trainees may graduate with the impression that they have learned psychotherapy even when psychotherapy is not a focus of their program. Though the prevalence of such beliefs is unknown and would require further research, mechanisms to address such misuse of training, such as guidelines about limits of competency, may be beneficial.

### Meeting the need for SERT-responsive care training

All interviewees acknowledged that SERT considerations were important for psychedelic care. This interest may represent a departure from other clinical fields, in which there is ambivalence about the role of religion or spirituality in treatment [50–52]. Despite the existence of standards and recommendations for SERT competence in medical and psychotherapeutic disciplines [53–55], best practices regarding SERT integration in psychedelics are still in formation [20]. Unlike many established practices, such as “set and setting” protocols [36,56], screening for contraindications to psychedelics [57], or the standardized phases of screening-preparation-dosing-integration [19,56,58], there is little agreement on explicit and shared guidelines for SERT-responsive care in psychedelics. Some programs expressed a need for SERT-integrated care training, while others were skeptical whether religious or spiritual content should be within the scope of facilitation training at all. Notwithstanding, all of the programs we spoke with affirmed the value of non-directive care that respected the spiritual and religious experiences, identities, histories, and practices of clients.

Programs that addressed SERT-related topics referred to a variety of frameworks for doing so, including transpersonal psychology, Jungian psychology, Integral philosophy, Internal Family Systems, Indigenous spirituality, and mindfulness. These are highly appropriate for some clients. However, to the extent that they imply unacknowledged SERT assumptions in the treatment model [59–61] they may represent a constrained and at times inappropriate palette of approaches to choose from. For example, as others have observed, models such as Jungian or transpersonal psychology can make universalist or perennialist assumptions that disregard the specificity and depth of variety of traditions, including those Indigenous traditions that have histories of psychedelics use [41,59,62,63]. Conversely, the wholesale application of specific approaches, such as those arising from Indigenous traditions, may harmfully appropriate knowledge without adequate expertise on the one hand, and be out of step with the cultural contexts and exigencies of non-Indigenous treatment contexts on the other. Some of these are “legacy” models, which have been integrated within psychedelic facilitation training due to their historic accompaniment of psychedelics’ emergence in the West. However, due to the underground nature of psychedelic practice, these have not benefitted from the same kind of disciplinary scrutiny as more-mainstream psychotherapeutic practices. Evidence-based and critically engaged strategies may provide useful tools for SERT-responsive psychedelic facilitation training.

For example, certified healthcare chaplains are well-prepared to care for patients from any and no religious background, with considerable focus on non-imposition and respect for patient autonomy in spiritual and religious domains [64,65]. Examining the theological or perspectival assumptions of routine care is a mainstay of chaplaincy training, offering tools that can be beneficially applied in psychedelics [30]. Drawing from training models like Clinical Pastoral Education [66], or including training for evidence-based spiritual health interventions, such as Compassion-Centered Spiritual Health [67], may offer viable strategies for supplementing existing psychedelic facilitation training. At well-resourced sites, facilitators may be in a position to refer or collaborate with spiritual health clinicians [68] or clergy [69], a strategy that exemplifies excellent standards of care. However, it is important to bear in mind that referrals to chaplains for SERT-related issues are not always feasible, even in clinical settings where established chaplaincy services operate, due to staffing and pragmatic limitations [70,71]. Importantly, such referrals are not a substitute for spiritually responsive care–indeed, therapeutic professions such as counseling and psychology have established religious and spiritual competencies because clinicians should have some degree of competency in such issues when they arise. Routine deferral of SERT issues to others may diminish quality of care [72] and potentially invalidate to patients who seek to discuss such issues with those clinicians actively offering care [73], and with whom they have established a relationship. Even outside of psychedelics, 56–75% of hospital patients [74], over half of outpatient psychotherapy clients [75], and 77–83% of older patients [76] have reported a desire for their therapists to address religion or spirituality in their therapeutic work.

Spiritually integrated psychotherapies offer another resource for SERT-responsive care training. Research and practice in spiritually integrated psychotherapies has produced an extensive empirical and conceptual literature to guide evidence-based care [77,78]. There exist spiritual and religious competence guidelines for counselors [53] and psychologists [55]. A broad repertoire of spiritually integrated therapeutics is available, and consistently shows non-inferiority or superiority to non-spiritually-integrated psychotherapies [77]. Although some spiritually integrated psychotherapies are related to specific denominations or traditions, several models apply a pluralistic stance toward religion, aiming to serve clients of any and no religion [6,79]. Training principles and techniques from such spiritually integrated psychotherapy models can be readily applied toward the education of SERT-competent psychedelic facilitators [20]. Notably, there seems to be openness to spiritually oriented psychotherapeutic models in psychedelic training, as might be inferred from the use of Jungian, Internal Family Systems, mindfulness, and transpersonal approaches, all of which take spirituality seriously. However, taking spirituality seriously does not in itself imply an evidence base of approaches to address spirituality and non-spirituality in its various forms, and indeed some of these approaches have been critiqued for insensitivity and inadequate responsiveness to different religious frameworks [80,81]. Lessons learned from spiritually-integrated psychotherapy approaches may be fruitfully integrated with models already applied in psychedelic contexts.

Scholarship in the academic study of religion has engaged and often challenged frameworks that are routinely invoked in psychedelic care, including the mystical experience as a model for spirituality [41,82], the centrality of personal experience in religious and spiritual transformation [83], or of perennial spirituality as a working model for religion [84]. If instruction about religion or spirituality in psychedelic care is insulated from these scholarly challenges, facilitators can fail to account for the wide diversity of SERT experiences. At worst, this can replicate colonial, ethnocentric, racist, and sexist assumptions that attend certain narratives on religion [85–87]

Opportunities to teach SERT-responsive care are limited by several structural constraints. These include lack of time to provide detailed training, lack of opportunities for continuing education, lack of clarity in state guidelines concerning SERT-responsive care, and lack of staff to teach expertly to all required content areas *and* SERT-responsive care. Programs often must balance between either only rudimentary SERT training, or omission of crucial material for those without a prior background.

Resolutions of these challenges should be harmonized with initiatives to address cross-cutting needs in psychedelic facilitator education. One way of doing so would be to adopt a continuing education model. Although there may not be time to provide extensive SERT-responsive care training within standard certification programming, specialized modules (comparable to continuing education requirements for other clinical disciplines) can allow greater focus on SERT-responsive care training while making clear the ongoing nature of training. Such modules can provide opportunities to gain instruction from experts with extensive experience teaching on SERT-integrated care. As is the case among disciplines like psychology, social work, and medicine, instructional workshops can offer continuing education credit to professionals from more than one field. Might psychedelic facilitation benefit from a similar model? Another potential strategy for addressing gaps in psychedelic training is through greater interdisciplinary collaboration, which may be extended to SERT-responsive care. Illustratively, public health and preventive medicine models have understood religion to be an important aspect of patients’ lives, and have included religious communities as part of a “collaborative continuum” of care [69] within which clinicians engage in interdisciplinary collaboration with religious professionals and paraprofessionals. Such collaboration may be highly beneficial for trainees and graduates of psychedelic facilitation programs. Indeed, even an exceptionally skilled spiritually integrated psychotherapist is aware that they are not a substitute for a clergy member or spiritual expert from a patient’s faith community and refers to those clergy at appropriate times. Patients’ lives are lived outside the clinic or psilocybin service center, and perhaps a final and crucial skill for any psychedelic facilitator is knowing when to pass the torch.

### Limitations

These findings should be interpreted in light of several important limitations. First, this was not a representative survey and therefore cannot speak to propensities or trends in a generalizable way. Instead, as a formative project that sought perspectives from a small but diverse set of training programs, its priority was to characterize different aspects of psychedelic facilitation training as described by the programs’ representatives. Our aims were to capture the perspectives of programs that occupied different positions on several variables: longevity/age; faculty and cohort size; location; training model; and intended applications. In focusing on these attributes, we of necessity sacrificed representativeness in the findings, a limitation that is appropriate to the QI nature of this project, but which must be overcome in subsequent research that is capable of more generalizable findings. Future research that can build on these findings to systematically assess and quantify the strategies and decisions implemented by training programs would be of great benefit.

Further, this project relied on a rapid, inductive-deductive approach to collecting and synthesizing data through interview notes. This introduces greater bias than traditional qualitative analysis, which was only partially mitigated by member-checking the interview summaries with interviewees. Another important limitation derives from the positionality and identity of the interviewers, as well as potential intellectual conflicts of interest they may hold. Specifically, the interviewers have previously published, worked in, and participated as trainees in the area of psychedelic facilitation. As a project initiated by the Center for the Study of World Religions at Harvard Divinity School, conducted by two interviewers who had previously published on the importance of SERT-integration in psychedelic care, the procedures were developed to learn information specifically germane to these areas and therefore relied on this specialized background. However, it is possible that interviewees attended to SERT issues more than they would have with other interviewers.

## Conclusion

Psychedelic facilitation training in the US is a dynamic and evolving landscape, contingent on policy, market forces, practice guidelines, and an interplay of traditions and norms. State regulatory guidelines meaningfully influence the nature, structure, and content of facilitation training programs, with many programs emphasizing the importance of safety, and of skills relevant to care seekers’ screening, preparation, dosing, and integration. And yet there does not appear to be ample time in training curricula to foster all requisite skills for psychedelic facilitators, a factor that has led programs to recruit applicants who already have foundational clinical or therapeutic skills, such that facilitation programs may best be understood as an important enhancement to existing competencies rather than stand-alone training. Despite unanimous attention to the importance of SERT-related phenomena in psychedelic care, SERT-responsive care training is inconsistent and frequently non-specific. This suggests the importance of ongoing, targeted training through continuing education modules and specialized modules that can attend to training priorities which cannot be adequately covered in typical training programs intended to meet requirements for state licensure.

## Supporting information

S1 This is the interview guide and participant information sheet used for this study. (DOCX)
