# Supplementary material for: A landscape analysis of psychedelic facilitation training in the US
Source: PLoS One. 2026 May 29;21(5):e0350037. doi: 10.1371/journal.pone.0350037 (PMC13220995; doi:10.1371/journal.pone.0350037)
Supplement: S1 — (DOCX) [file pone.0350037.s001.docx]

Thank you for taking part in this interview. We are speaking with you because we believe you have an important role in the developing field of psychedelic facilitation training.

We are interested in learning more about the landscape of psychedelic facilitation training in the US. This is not research, but rather information-gathering from key stakeholders in our developing field.

There are many phenomenal training programs in the US. Because the needs for rigorous training are only increasing, we are hoping to better understand the priorities, practices, and pedagogical emphases of high-quality programs such as yours. This will result in a public-facing report hosted by Harvard’s Center for the Study of World Religions, but which would be available free for all. Ideally, any group, program, or entity would be able to use the results of this project however they see fit. In the report we'd like to elevate what seems to be working and relative strengths you draw on in your program, as well as any recognized gaps in training. This is not evaluative: our purpose is to recognize approaches that work, and specific needs that are either common across programs, or which are unique to specific contexts.

We are inviting you to a conversation with Caroline Peacock and Roman Palitsky. This conversation would be recorded if you approve, and we would organize and synthesize the information that we gather from you. We won't retain the recording or make it available to anyone. We would also send the synthesis back to you for approval, revision, or clarification: we want to make sure that your perspectives are represented well.

Below is a description of the topics we will be asking about. The conversation is meant to be flexible and adaptive to the areas and topics that you believe to be particularly important, rather than comprehensive and exhaustive.

We’re also happy to answer any questions prior to, or during, our meeting. Please reach out with any questions or concerns (our contact information is below). Thank you for considering taking part in this project, and for your response. We look forward to hearing from you.

Sincerely,

Roman Palitsky, MDiv, PhD ([roman.palitsky@emory.edu](mailto:roman.palitsky@emory.edu), 646 667 8250)

Caroline Peacock, DMin, LCSW, BCC, ACPE ([caroline.peacock@emory.edu](mailto:caroline.peacock@emory.edu), 404 778 8029)

We will be asking you about the following topics:

**General information: what is the scope and focus of the program?**

What psychedelics, if any, does your program specialize in?

Who are your target/optimal trainees? (e.g., what qualifications do they need to have?)

What background or preparation do your faculty need to have?

What level of autonomy are your trainees expected to have post-training? Is there a certification/licensure available through your program?

How long has your program been active?

**What is your approach to facilitator competencies? Which competencies do you believe are particularly important, and have you identified good ways to instill those competencies in your trainees?**

Here are some examples, but this is by no means a comprehensive list:

- Cultural competence
- spiritual, existential, religious, and theologically-responsive care
- competence with non-ordinary states
- use of music
- trauma-sensitive care & safety building
- biopsychosocial assessment
- use of touch
- therapeutic techniques
- therapeutic presence; preparation skills
- integration skills
- ethical discernment
- interprofessional collaboration
- continuing education, consultation, supervision

**What is your approach to training in the prevention and addressing of adverse events, challenges, or persisting distress among the trainees’ clients?**

**How is your program structured?**

This pertains to a number of structural considerations, which include but are not limited to considerations about:

- time (e.g., number of meetings, hours, duration of the program)
- space (e.g., location, locality)
- resources (e.g., financial, intellectual)
- personnel (e.g., faculty, staff, faculty/trainee ratios)
- format (e.g., in-person, experiential components)
- finances (e.g., fees, scholarships, economic sustainability for your program and for trainees)
- selection (e.g., admissions rates, selection priorities, application processes)
- sustaining relationships with other institutions (e.g., university, non-profit, treatment center, or other entities)

What is your program’s approach to supporting access, equity, and inclusion of diverse and under-represented trainees and faculty?

**What pedagogical approaches do you favor?**

Is there a pedagogical philosophy or philosophies that inform your program? How do you evaluate training success? What is your approach to use of non-ordinary state experiential components for your trainees?

**What is your approach to professionalization in the psychedelic care field?**

Professionalization may involve parity with other programs and recognition of one another’s training certifications, participation in a network with other programs, or other involvement in a broader profession or field.

This may also involve:

- Training-specific conferences, pre-conferences, or professional meetings for exchange of ideas about psychedelic training
- Tracking whether, and where, graduates are practicing
- Engagement with existing professions, boards, and their standards
- Accountability to standards embraced by existing professions, such as psychology, nursing, social work, etc.
- Relationships with groups or networks of practice and knowledge, including Indigenous rights-holders, underground networks, etc.

**What is your stance toward evidence-based practice?** This can include evidence-based pedagogy, teaching empirically supported techniques and tools, etc. What counts as evidence-based?

**What is your program’s approach to reciprocity with the Indigenous communities, with the natural environment, and with the community in which your program is located?**

**What are your unmet needs, growth goals, or other desirable elements you would like to see happen for your program or the field more generally?**
